# Ectopic ACTH Production Leading to Diagnosis of Underlying Medullary Thyroid Carcinoma

**DOI:** 10.1177/2324709616643989

**Published:** 2016-04-08

**Authors:** Leslee N. Matheny, Jessica R. Wilson, Howard B. A. Baum

**Affiliations:** 1 Vanderbilt University Medical Center, Nashville, TN, USA

**Keywords:** ectopic ACTH secretion, Cushing’s syndrome, medullary thyroid carcinoma

## Abstract

Medullary thyroid carcinoma (MTC) has been described as a source of ectopic ACTH secretion in patients with Cushing’s syndrome. This is an infrequent association, occurring in less than 1% of MTC cases. Among these, it is even more unusual for an initial diagnosis of hypercortisolism to lead to the discovery of underlying MTC. Here we present a case of a patient with weakness, diarrhea, and hypokalemia who was found first to have Cushing’s syndrome and later diagnosed with metastatic MTC. The patient was treated initially with oral agents to control his hypercortisolism, then with an etomidate infusion after experiencing intestinal perforation. He also received vandetanib therapy targeting his underlying malignancy, as this has been shown to reverse clinical signs of Cushing’s syndrome in patients with MTC and subsequent ectopic ACTH secretion. Bilateral adrenalectomy was ultimately required. Medullary thyroid carcinoma should be considered in patients presenting with Cushing’s syndrome due to ectopic ACTH secretion, and a multimodality treatment approach is often required.

## Introduction

Medullary thyroid carcinoma (MTC) is a malignancy derived from the calcitonin-producing parafollicular C cells of the thyroid gland. It accounts for up to 1% to 2% of all thyroid cancers and includes both sporadic and familial forms.^1^ The inherited cases are typically associated with germ-line mutations in the RET proto-oncogene, and may coexist with other neoplasia as part of the multiple endocrine neoplasia type 2 (MEN2) familial syndromes. Patients diagnosed with MTC typically have a poorer prognosis than those with other forms of differentiated thyroid cancer. Factors such as age over 40 years, male gender, the presence of metastases at time of diagnosis, and the presence of a paraneoplastic syndrome all negatively affect survival.^2^ Patients with tumors confined to the thyroid gland have been found to have a 10-year survival rate of 95.6%, compared to 40% in patients with distant metastases at the time of diagnosis.^3^

While these tumors secrete calcitonin, it is uncommon for them to be associated with a paraneoplastic syndrome. In a retrospective review of 1640 cases of MTC, at most 0.7% developed ectopic ACTH secretion.^2^ Of these cases, it has been estimated that the underlying MTC is discovered first in 65% of patients.^4^ Here, we present a case of a patient diagnosed initially with Cushing’s syndrome who was subsequently found to have metastatic MTC as the source of excess ACTH. This highlights the importance of considering thyroid carcinoma as the driver of excess cortisol in patients who present with ectopic Cushing’s syndrome. His degree of hypercortisolemia became severe, requiring an etomidate infusion while also treating underlying infection prior to bilateral adrenalectomy.

## Case Presentation

A 67-year-old man presented with 4 months of progressive diarrhea and weakness. Medical history was remarkable for hypertension, mycosis fungoides, diverticular disease, hepatic and renal cysts, and a history of colonic surgery for polyp removal. Physical exam was notable for a palpable lymph node in the left lateral neck without a discernable thyroid mass, bilateral lower extremity edema, and proximal muscle weakness of all extremities. Initial laboratory evaluation revealed hypokalemia to 2.8 mmol/L (3.3-4.8), a new diagnosis of diabetes with hemoglobin A1C 6.8%, elevated hepatic enzymes with aspartate transaminase 1.1 µkat/L (0.08-0.67) and alanine transaminase 2.29 µkat/L (0-0.92), and abnormal thyroid testing with thyroid-stimulating hormone (TSH) 0.144 mIU/L (0.35-3.60) and FT4 8.11 pmol/L (9.01-17.63).

Follow-up testing obtained due to his profound myopathy and hypokalemia noted an elevated ACTH of 69.08 pmol/L (1.54-11.22), random serum cortisol 1453.89 nmol/L (102.08-535.21), serum sodium 151 mmol/L (136-144), and potassium 2.6 mmol/L, prompting hospitalization. Subsequent 24-hour urine free cortisol was 11 119 nmol/day (<165.6). Serum cortisol did not suppress to low- or high-dose dexamethasone suppression testing; serum cortisol was 1448.37 and 1431.82 nmol/L after 1 mg and 8 mg of dexamethasone, respectively. Aldosterone and renin levels were normal. Pituitary magnetic resonance imaging revealed a 4 mm, relatively hypoenhancing lesion in the right inferior aspect of the gland on gadolinium-contrasted images. No effect on the pituitary stalk or optic chiasm was present, and there was no invasion of the cavernous sinus.

**Figure 1.**
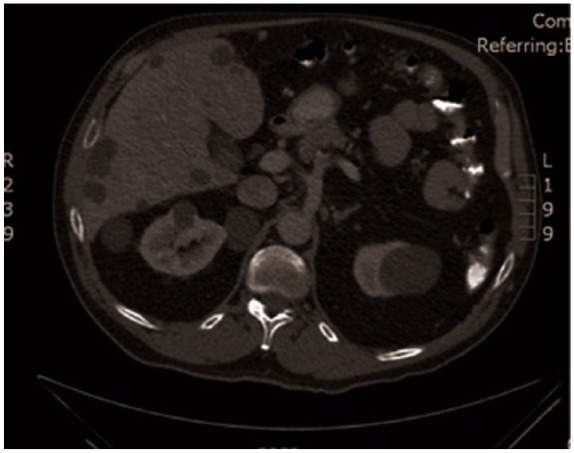
CT abdomen/pelvis with contrast 3 months prior to presentation, obtained in the setting of diverticular disease. Innumerable low-attenuation liver and multiple kidney cysts noted previously.

**Figure 2.**
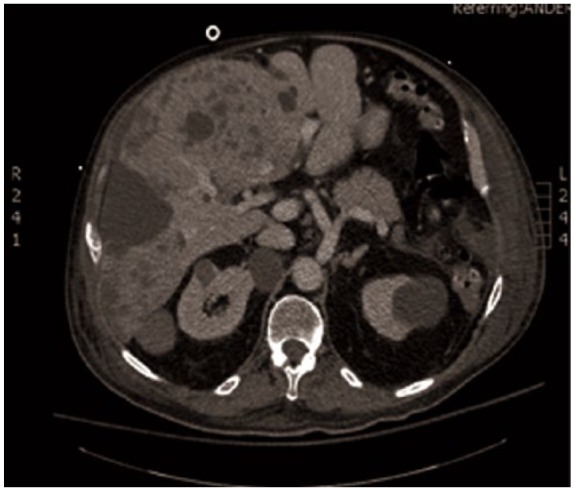
CT abdomen/pelvis with contrast obtained for evaluation of hypercortisolism. Note the presence of new low-density liver lesions not seen 3 months prior, consistent with metastases. Also, new bilateral adrenal hyperplasia.

**Figure 3.**
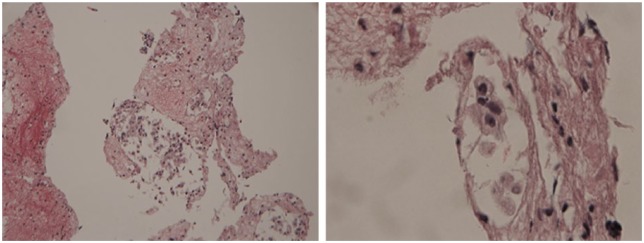
Fine needle aspiration of liver lesion at low power (left) and high power (right) showing involvement with medullary thyroid carcinoma. Hematoxylin and eosin stain.

**Figure 4.**
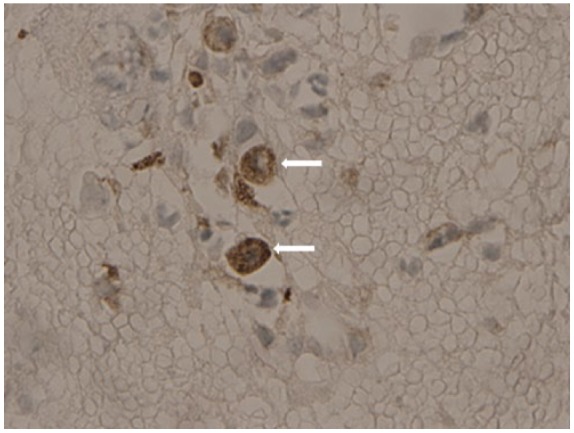
Fine needle aspiration of liver lesion, calcitonin immunostaining positive (arrows). Consistent with metastatic medullary thyroid carcinoma.

Due to his degree of cortisol excess and failure to suppress after high-dose dexamethasone, further evaluation for ectopic ACTH secretion was performed. This included normal levels of vasoactive intestinal polypeptide and 5-hydroxyindoleacetic acid, but calcitonin returned elevated at >5840 pmol/L (0-3.36), as did chromogranin A at 1089 µg/L (<96). Inferior petrosal sinus sampling was considered clinically unnecessary. Computed tomography (CT) scan of the chest and abdomen with contrast showed new adrenal hypertrophy and low-density liver lesions not seen 3 months prior (Figures 1 and 2). Given the elevated calcitonin level, thyroid imaging was pursued and revealed an irregular 2 cm left thyroid mass and lymphadenopathy. Left neck lymph node and liver biopsies confirmed the diagnosis of metastatic MTC at both sites (Figures 3 and 4).

Initial therapy for his hypercortisolism included ketoconazole and spironolactone, which normalized his serum potassium, although serum cortisol remained elevated >1300 nmol/L. Ketoconazole was discontinued due to hepatic enzyme elevation, and mifepristone was initiated instead. Despite this, he developed progressive weakness, abdominal pain, and a perforated diverticulum with associated intra-abdominal abscess, which was treated with percutaneous drainage and intravenous antibiotics. Due to his rapid clinical deterioration, mifepristone therapy was stopped after 5 days, and he was then treated with etomidate (2.5-3.5 mg/h infusion) and hydrocortisone for 16 days prior to undergoing laparoscopic adrenalectomy. Serum cortisol levels with concurrent etomidate and hydrocortisone therapy improved to goal of 550 to 800 nmol/L, in the setting of acute illness. He was also administered vandetanib therapy for metastatic MTC for 11 days (including 5 days postoperatively). Adrenal pathology was negative for malignancy. Despite this, his clinical status continued to decline, progressing to obtundation requiring mechanical ventilation. He received antibiotics for possible pneumonia without improvement, and life-support measures were subsequently withdrawn on postoperative day 8. His family declined a request for autopsy.

## Discussion

Endogenous Cushing’s syndrome is characterized by excessive glucocorticoid production, which is ACTH-dependent in 80% of cases and usually due to a pituitary corticotroph adenoma (Cushing’s disease).^5^ In approximately 20% of patients, ACTH is produced ectopically by nonpituitary tumors, with small cell lung cancers; thymic, pulmonary, and pancreatic carcinoid tumors; pheochromocytomas; and MTCs being the most common tumor types.^6^ It is estimated that MTC is the source of ACTH secretion in 2% to 8% of ectopic Cushing’s syndrome cases.^7^

Approximately 50 cases of Cushing’s syndrome due to MTC have been reported. Of these, it is unusual for the diagnosis of hypercortisolism to precede that of thyroid cancer, as occurred in our patient. In one review, only 3 of 10 patients with MTC presented first with ectopic ACTH production, which corresponded to previously published data.^2^ Thus, tumors may acquire the ability to produce ACTH at any time, and cases have even been reported more than 20 years after initial MTC diagnosis.^8^

When Cushing’s syndrome does occur, the diagnosis may be challenging. In cases of ACTH-dependent disease, it is sometimes difficult to distinguish between pituitary and nonpituitary sources. This is in part because some patients with ectopic ACTH secretion may still respond to high-dose dexamethasone suppression testing, making their biochemical profile appear consistent with a central source. In one retrospective review of patients ultimately diagnosed with ectopic sources of ACTH, positive responses to dexamethasone were seen in up to 14%.^9^ This occurred most frequently in the population of patients with pulmonary carcinoid tumors.

The diagnosis is further complicated by the prevalence of nonfunctioning pituitary microadenomas. It has been suggested in a consensus statement that a pituitary magnetic resonance imaging should be performed in all patients with ACTH-dependent hypercortisolism, keeping in mind that 10% of the general population will harbor incidental pituitary tumors.^10^ As the majority of these lesions are less than 5 mm in diameter, expert opinion suggests that in patients with a classic clinical presentation and biochemical studies compatible with pituitary Cushing’s, using a cutoff >6 mm for a focal pituitary lesion may obviate the need for inferior petrosal sinus sampling (IPSS) to diagnose Cushing’s disease.^10^ Our patient did have a 4 mm pituitary lesion, though due to its small size, the clinical context, the degree of ACTH elevation and hypokalemia, and inability to suppress serum cortisol after high-dose dexamethasone, IPSS was not pursued.

Also of interest is that our patient had thyroid function tests showing both low TSH and FT4, consistent with central hypothyroidism. It has been noted that hypercortisolism can suppress thyroid function, probably through inhibition of thyroid releasing hormone and TSH secretion, as well as suppression of the 5′-deiodinase enzyme that converts T4 into active T3.^10^

Regardless of source, when Cushing’s syndrome does develop, the treatment of choice is surgical resection of the offending lesion. In cases where the tumor cannot be found or surgery is not successful or not possible, medical therapy to control the excess cortisol production is a necessity. This may take several forms, including the steroidogenesis inhibitors ketoconazole and metyrapone, the glucocorticoid receptor blocker mifepristone, or the adrenolytic agent mitotane. In cases of Cushing’s disease, pituitary-directed therapies such as the somatostatin analog pasireotide and the dopamine agonist cabergoline are also available.^5^ However, with the exception of pasireotide, all of these therapies are oral, and may not be feasible in times of critical illness or in the face of intestinal perforation, as was the case with our patient. This becomes critical as perforation is a known complication of Cushing’s syndrome and the cause of death in 30% of MTC cases with ectopic ACTH secretion.^2^ Furthermore, therapy with ketoconazole may be limited by hepatic enzyme elevation, which our patient also experienced.

In situations where an intravenous medication is preferred or when rapid control of hypercortisolism is necessary, etomidate may serve as an alternative treatment. Etomidate, like ketoconazole, is a member of the imidazole family, and blocks cortisol synthesis by inhibiting the adrenal enzyme 11B-hydroxylase, which catalyzes the conversion of deoxycortisol to cortisol. There is a wide dosing range between adrenostatic and anesthetic effects, and it has been found that infusion at lower doses of 0.01 to 0.1 mg/kg/h is effective at inhibiting adrenal steroidogenesis within hours.^11^ A “block and replace” strategy has been suggested, targeting a cortisol level that would be expected in a given clinical context in the absence of Cushing’s syndrome.^12^ Replacement therapy with concurrent administration of glucocorticoid is then required.

Notably, our patient also received therapy with vandetanib, which is the first drug approved in the United States for treatment of symptomatic and/or progressive MTC in patients with unresectable, locally advanced, or metastatic disease.^13^ It is a once daily, oral tyrosine kinase inhibitor that has been reported in 3 cases (2 adult and 1 pediatric) to improve serum cortisol levels and reverse clinical signs of Cushing’s syndrome due to ectopic ACTH secretion from the thyroid malignancy.^13-15^ In 2 of the cases, the cortisol levels normalized completely.^14,15^ In none, however, did vandetanib reduce tumor burden.

## Conclusion

In this case, a patient was diagnosed with ectopic ACTH production, leading to the diagnosis of underlying medullary thyroid carcinoma. MTC alone portends a poorer prognosis than other differentiated thyroid cancers, even more so when a paraneoplastic syndrome is present. This is an uncommon entity, with only about 50 cases reported in the literature. This case highlights the importance of considering MTC as a source of ACTH in patients with ectopic Cushing’s syndrome. Due to the severity of his hypercortisolemia and critical illness in the setting of a bowel perforation, the need to control infection acutely, and also medication limitations in the setting of hepatic enzyme elevation, our patient was treated with an etomidate infusion and vandetanib to decrease cortisol production.
